# Identification of MMP1 as a potential gene conferring erlotinib resistance in non-small cell lung cancer based on bioinformatics analyses

**DOI:** 10.1186/s41065-020-00145-x

**Published:** 2020-07-23

**Authors:** Huyue Zhou, Qiumei Xiang, Changpeng Hu, Jing Zhang, Qian Zhang, Rong Zhang

**Affiliations:** 1 Department of Pharmacy, the Second Affiliated Hospital of Army Medical University, 83 Xinqiao Road, Chongqing, 400037 China; 2 Maternity service center of Beijing Fengtai District Maternal and Child health care hospital, Beijing, 100067 China

**Keywords:** Bioinformatics analyses, NSCLC, Erlotinib resistance, MMP1

## Abstract

**Background:**

Non-small cell lung cancer (NSCLC) is the major type of lung cancer with high morbidity and poor prognosis. Erlotinib, an inhibitor of epidermal growth factor receptor (EGFR), has been clinically applied for NSCLC treatment. Nevertheless, the erlotinib acquired resistance of NSCLC occurs inevitably in recent years.

**Methods:**

Through analyzing two microarray datasets, erlotinib resistant NSCLC cells microarray (GSE80344) and NSCLC tissue microarray (GSE19188), the differentially expressed genes (DEGs) were screened via R language. DEGs were then functionally annotated by Gene Ontology (GO) analysis and Kyoto Encyclopedia of Genes and Genomes (KEGG) analysis, which up-regulated more than 2-folds in both datasets were further functionally analyzed by Oncomine, GeneMANIA, R2, Coremine, and FunRich.

**Results:**

We found that matrix metalloproteinase 1 (MMP1) may confer the erlotinib therapeutic resistance in NSCLC. MMP1 highly expressed in erlotinib-resistant cells and NSCLC tissues, and it associated with poor overall survival. In addition, MMP1 may be associated with COPS5 and be involve in an increasing transcription factors HOXA9 and PBX1 in erlotinib resistance.

**Conclusions:**

Generally, these results demonstrated that MMP1 may play a crucial role in erlotinib resistance in NSCLC, and MMP1 could be a prognostic biomarker for erlotinib treatment.

## Introduction

Lung cancer is the primary cancer-related death worldwide [1, 2]. Non-small cell lung cancer (NSCLC) is the main type of lung cancer, including squamous cell carcinoma, adenocarcinoma, and large cell carcinoma, which accounts for approximately 85% of all cases [3–5]. Currently, chemotherapy plays a crucial role in NSCLC treatment for most patients missing the optimal surgical timing or with distant metastases [6]. Platinum-based traditional drug chemotherapy is the standard first-line therapy for patients with advanced lung cancer, which improved the clinical outcomes modestly [7]. However, it causes severe side effects, including nephrotoxicity, neurotoxicity, and emetogenic effect, which usually resulted in a poor quality of life [8]. In recent years, molecular-targeted therapy has been developed into the most promising strategy for NSCLC treatment, which exerts greater anticancer efficacy and fewer side effects than existing chemotherapy [9]. Erlotinib is a specific inhibitor of epidermal growth factor receptor (EGFR) tyrosine kinase, which is given for locally advanced or metastatic non-small cell lung cancer treatment [10–12]. It can block EGFR downstream signaling pathways such as the signal transducer and activator of transcription 3 (STAT3) and mitogen-activated protein kinase (MAPK) pathway [13]. Although erlotinib has been confirmed to improve the prognosis of NSCLC patients, the majority of these patients inevitably develop acquired resistance with the prolonged treatment time [14, 15]. Several mechanisms of erlotinib resistance have been identified, including T790M gatekeeper EGFR-mutation, activation of bypass-signaling (amplification of IGF1R and MET) [16–20]. Besides, 40–60% of acquired resistance mechanism is EGFR T790M mutation in first generation EGFR-TKIs [21]. However, the erlotinib acquired resistance mechanisms for NSCLC patients without T790M mutation is not clear.

Genomics microarray, a high-throughput platform, represents a great powerful technology to analyze gene expression comprehensively [22, 23]. In combination with multiple bioinformatics analyses, microarray technology has been widely valued as a tool with great promise to obtain gene signals during tumor process, drug resistance, and verify prognostic biomarkers in cancers [24–26]. Detection and verification of vital targets of drug resistance by using microarray analysis is a novel research strategy and may in favor of uncovering the underlying mechanisms of drug resistance.

Here, we acquired NSCLC of erlotinib resistance and sensitive microarray (GSE80344) and NSCLC adjacent and tumor tissue microarray (GSE19188) from the Gene Expression Omnibus (GEO) online database (https://www.ncbi.nlm.nih.gov/geo/), and these microarrays were analyzed by bioinformatics methods. To functionally annotate the differentially expressed genes (DEGs), we employed the Gene Ontology (GO) analysis and Kyoto Encyclopedia of Genes and Genomes (KEGG) analysis. Moreover, we further functionally analyzed the genes which up-regulated more than 2-folds in both datasets by Oncomine, GeneMANIA, R2 Coremine, and FunRich to identify that MMP1 was a crucial gene of erlotinib resistance in NSCLC. Meanwhile COPS5 was related to MMP1, and both of their high expressions affected patients’ survival. Also, transcription factors HOXA9 and PBX1 may play an important role in this process. Our study provides a potential target and associated mechanisms of erlotinib resistance and suggests that MMP1 could be a prognostic biomarker to erlotinib treatment.

## Materials and methods

### Microarray data

Two high-throughput sequencing and expression profiling microarray datasets were obtained from the GEO database (https://www.ncbi.nlm.nih.gov/geo). In detail, GSE80344 (*n* = 16) is composed of 12 erlotinib-resistant and 4 erlotinib-sensitive samples in NSCLC and GSE19188 (*n* = 156, 3 datasets were abandoned) consists of 91 human NSCLC and 62 adjacent normal tissues. The platforms are GPL16699 and GPL570, respectively.

### Processing of microarray data

The matrix files were downloaded using the R language, and the adjacent and tumor tissue gene expression profiles (GSE19188) were analyzed using the Lima software package to obtain DEGs. DEGs in the NSCLC erlotinib-resistant gene expression profiles (GSE80344) were subsequently analyzed using GEO2R (https: / /www.ncbi.nlm. nih.gov/geo/geo 2r/). GEO2R is an online tool to compare two or more groups of samples to identify genes which differentially expressed across experimental conditions. The DEGs cut-off criteria were set to *P* values < 0.01 and |log_2_FC| ≥ 2 (differential expression multiples ≥4), where log_2_FC ≥ 2 is upregulated, log_2_FC ≤ − 2 was downregulated, and the error rate was reduced using the Benjamini & Hochberg method. And a heatmap of the DEGs was produced using Heml 1.0.3.7 heatmap illustrator software and it showed the top 50 upregulated and downregulated genes, respectively. All significant DEGs were shown in a volcano plot produced by R software.

### Functional and pathway enrichment analyses

Gene ontology analysis is a generally used method for biological functional studies of high-throughput genomics information and transcriptome data [27]. It primarily annotates three ontologies: biological process (BP), cellular component (CC), and molecular function (MF). Kyoto Encyclopedia of Genes and Genomes (KEGG) stores a large amount of data on gene interactions and is commonly used in the signal pathway analysis [28]. To perform functional annotations on the up-expression DEGs, we subsequently conducted the Database, Visualization and Integrated Discovery (DAVID, https://david.abcc.ncifcrf.gov/), to process the gene ontology and Kyoto Encyclopedia of Genes and Genomes (KEGG) signal pathway analysis. As a result, *P* < 0.01 was considered statistically significant.

### Expression level analysis

The erlotinib sensitive and resistant NSCLC dataset GSE38121, GSE69181 and GSE80344 were obtained from the GEO database (https://www.ncbi.nlm.nih.gov/geo), and analyzed by GEO2R (|log_2_ (Fold change) | > 1 and *P* < 0.01). And the expression data (GSE7670 and GSE10072) of MMP1 in normal tissues and tumor tissues were obtained by the online analysis website Oncomine (https://www.oncomine.org/resource/login.html). In detail, GSE7670 (*n* = 57) is composed of 30 normal lung tissues and 27 lung adenocarcinoma tissues, and GSE10072 consists of 49 normal lung tissues and 58 lung adenocarcinoma tissues. And the screening conditions were |log_2_ (Fold change) | > 2 and *P* < 0.0001. To confirm the expression level of MMP1, GEPIA2 online database was applied based on TCGA and GTEx data. The lung adenocarcinoma (LUAD) and lung squamous cell carcinoma (LUSC) datasets were selected, and genes with |log_2_ (Fold change) | > 2 and *P* < 0.01 were considered significant. We also analyzed the protein expression of the hub genes, COPS5, between NSCLC and normal lung tissues using the Human Protein Atlas (HPA, https://www.proteinatlas.org) database.

### Survival analysis

The NSCLC patients were respectively separated into high expression group and low expression group according to MMP1 expression levels, Kaplan-Meier plotter (KM plotter, https://www.kmplot.com) was used to estimate the effect of MMP1 on the survival of patients [29]. The dataset GSE50081 is composed of 127 lung adenocarcinoma patients, and GSE31210 consists of 226 lung adenocarcinoma patients. And the method excluding biased arrays was adopted to control array quality. In the GEPIA2 online database (http://gepia2.cancer-pku.cn/), the survival analysis was performed based on 144 lung adenocarcinoma (LUAD) patients’ data of a multi-gene signature (MMP1 and COPS5).

### Analyses of MMP1 interactions

Functional network predictive analysis of MMP1 was performed using the GeneMANIA (https://www.genemania.org/), which is an online gene functional analysis software used to generate hypotheses of gene function, analyze gene lists, and prioritizes genes for functional assays [30]. After entering MMP1 into the search bar and selecting humans from optional organisms, the results show 20 genes closely related to MMP1. At the same time, the R2 tool was used to analyze the correlation of proteins interacting with MMP1. In addition, the biological processes of MMP1, erlotinib, and drug resistance (neoplasm) were further annotated via consulting the Coremine Medical online Database (https://www.coremine.com/medical/).

### Identification of transcription factors targeting MMP1

FunRich (https://www.funrich.org) is a stand-alone software tool used mainly for interaction network analysis of genes and proteins [31]. In this study, we predicted and analyzed the transcription factors targeting MMP-1 by using FunRich.

### Statistical analysis

The GSE80344 data set was processed using the statistical method Benjamini & Hochberg (false discovery rate, FDR) in GEO2R, and the differential genes were screened using the SAM method. The two groups were compared by the two-tailed Student’s T-test. The difference was considered statistically significant at *P* < 0.05. GO enrichment analysis and KEGG signaling pathway analysis were performed using statistical tools provided by the DAVID database, adopting the Bonfeerrni, Benjamini false discovery rate, Bootstrap, and Fisher’s exact test. All the statistical analyses were conducted with the R language and SPSS 22.0 software.

## Results

### Identification and functional characterization of Upregulated DEGs in Erlotinib resistant NSCLC cells

**Fig. 1:**
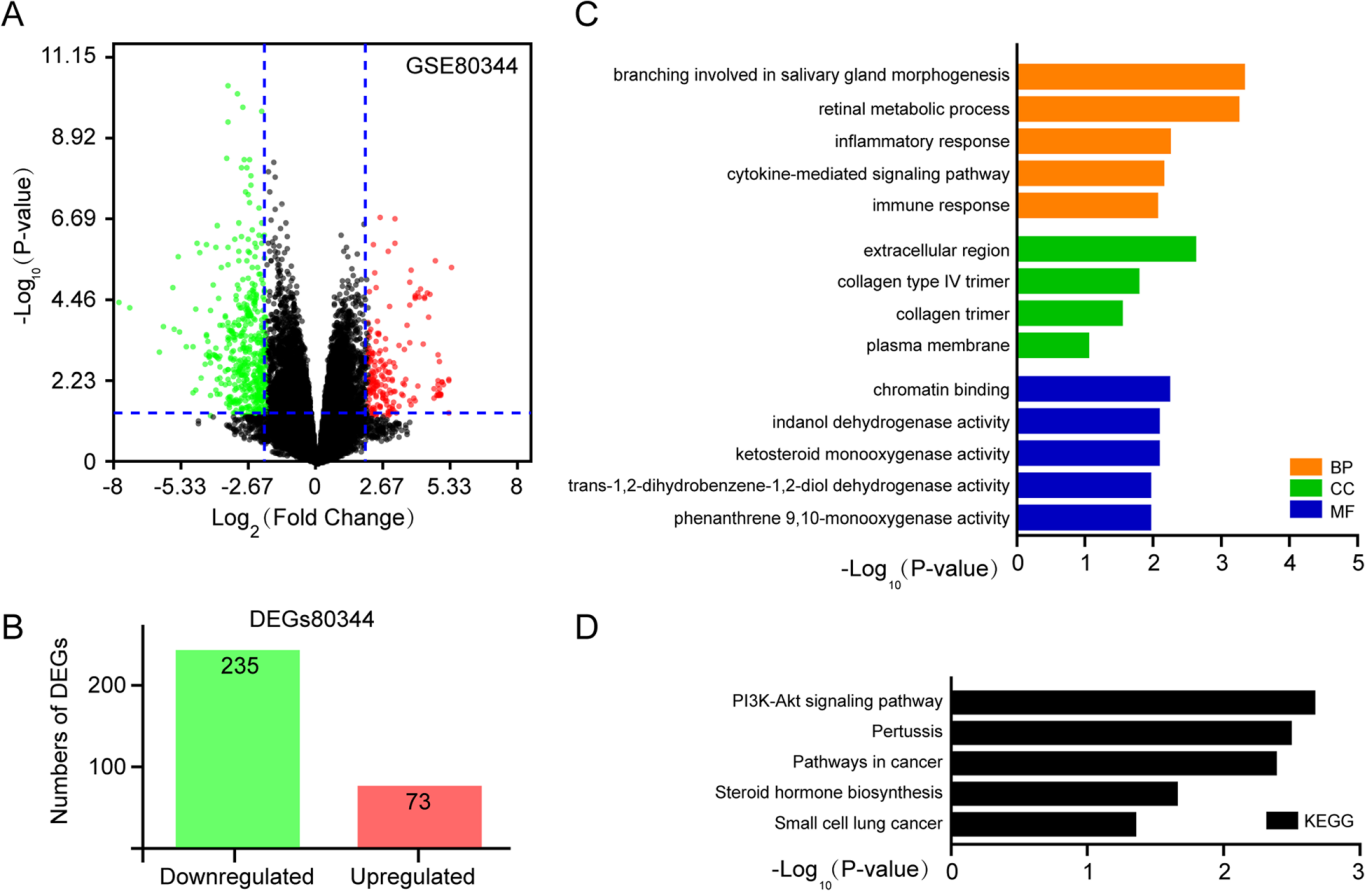
Identification and functional characterization of DEGs from GSE80344 dataset. **a** The DEGs volcano plot was analyzed between erlotinib resistant NSCLC cell lines and erlotinib sensitive cell lines. Green dots were significantly downregulated DEGs, red dots were significantly upregulated DEGs, and black dots were no significant difference (*P* < 0.01 and |log_2_FC| ≥ 2 as the threshold cutoff). **b** The distribution of significant DEGs in erlotinib resistant cells. The number of the upregulated genes was 73 and the downregulated genes was 235. The top 5 GO (**c**) and KEGG pathways (**d**) of significantly upregulated DEGs were indicated. MF, molecular function; CC, cellular component; BP, biological process

Erlotinib, an epidermal growth factor receptor (EGFR) tyrosine kinase inhibitor, is an oral targeted anticancer drug for non-small cell lung cancer (NSCLC) treatment, and showing a significant improvement of survival in NSCLC [32]. However, erlotinib-acquired resistance is a tough obstacle to effectively treating NSCLC patients with EGFR mutant characteristics [33–35]. The mechanism underlying erlotinib resistance in NSCLC has not been fully investigated. In this study, we first explored the potential genes conferring erlotinib resistance in the NSCLC erlotinib resistance and sensitive microarray (GSE80344). 308 DEGs in this microarray were identified (*P* < 0.01 and |log_2_FC| ≥ 2 as the threshold cutoff) and a volcano map was shown in Fig. 1a. Among these DEGs, there were 73 significantly upregulated and 235 markedly downregulated (Fig. 1b; Table S1). To characterize the potential role of these significantly upregulated DEGs, these DEGs were first performed for a GO enrichment analysis, results were divided into three ontologies, including biological process (BP), cellular component (CC) and molecular function (MF), and the top five GO enrichment terms of each ontology were shown in Fig. 1c; Table S2**.** In the BP category, upregulated DEGs were enriched in branching involved in salivary gland morphogenesis, retinal metabolic process, inflammatory responses, cytokine-mediated signaling pathway, and immune response. In the CC category, the upregulated genes were mainly enriched in the extracellular region, collagen type IV trimer, collagen trimer, and plasma membrane. In the MF category, the DEGs were enriched in chromatin binding, indanol dehydrogenase activity, ketosteroid monooxygenase activity, trans-1,2-dihydrobenzene-1,2-diol dehydrogenase activity, and phenanthrene 9,10-monooxygenase activity. Moreover, KEGG pathway enrichment analyses were further employed to investigate the functional annotations of these upregulated DEGs. Results showed that these upregulated DEGs were primarily involved in the PI3K-Akt signaling pathway, pertussis, pathways in cancer, steroid hormone biosynthesis, and small cell lung cancer (Fig. 1d; Table S3).

### MMP1 was identified as the gene conferring Erlotinib resistance in NSCLC

**Fig. 2:**
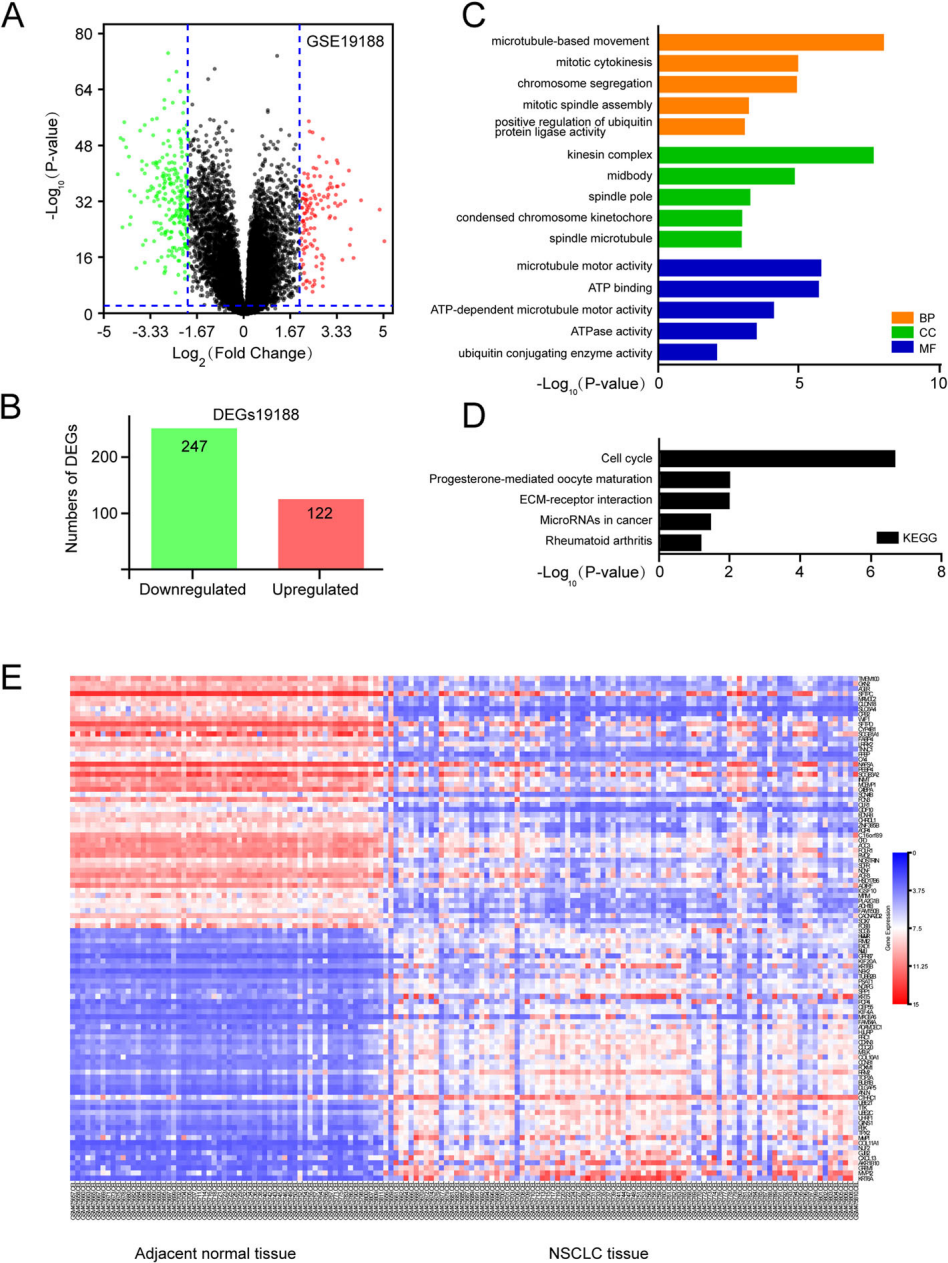
Identification and characterization of DEGs from GSE19188 dataset. **a** Volcano plot of DEGs between NSCLC tissues and the adjacent normal tissues. Green dots, significantly downregulated DEGs in NSCLC tissues; red dots, significantly upregulated DEGs in NSCLC tissues; black dots, no significant difference (*P* < 0.01 and |log_2_FC| ≥ 2 as the threshold cutoff). **b** The distribution of significant DEGs in NSCLC tissues. The number of the upregulated genes was 122 and the downregulated was 247. The top 5 GO (**c**) and KEGG pathways (**d**) of significantly upregulated DEGs were indicated. **e** Heatmap of top 50 upregulated and downregulated DEGs. Red, upregulation; blue, downregulation. MF, molecular function; CC, cellular component; BP, biological process

Whether these upregulated DEGs in the erlotinib-resistant GSE80344 dataset are also highly expressed in other NSCLC datasets, we further analyzed a dataset GSE19188 (*n* = 156, 3 datasets were abandoned) consisting of 91 human NSCLC and 62 adjacent normal tissues. A total of 369 DEGs in dataset GSE19188 were identified by R language analysis with *P* < 0.01 and threshold cutoff |log_2_FC| ≥ 2, a volcano map was shown in Fig. 2a. Among these DEGs, 122 genes were dramatically upregulated and 247 genes were downregulated (Fig. 2b; Table S4). These upregulated DEGs were further functionally annotated by GO analyses, and the top five GO enrichment terms of BP, CC, and MF categories were shown in Fig. 2c; Table S5. In the BP category, upregulated DEGs were enriched in microtubule-based movement, mitotic cytokinesis, chromosome segregation, mitotic spindle assembly, and positive regulation of ubiquitin-protein ligase activity. In the CC category, the upregulated genes were mainly enriched in kinesin complex, midbody, spindle pole, condensed chromosome kinetochore, and spindle microtubule. In the MF category, the upregulated DEGs were enriched in microtubule motor activity, ATP binding, ATP-dependent microtubule motor activity, ATPase activity, and ubiquitin-conjugating enzyme activity. In addition, KEGG analysis results showed that these upregulated DEGs were mainly involved in signaling pathways of the cell cycle, progesterone-mediated oocyte maturation, ECM-receptor interaction, microRNAs in cancer and rheumatoid arthritis (Fig. 2d; Table S6). Expanded view of the expression of top 50 upregulated and downregulated genes of NSCLC tissues was shown in heatmap (Fig. 2e).

**Fig. 3:**
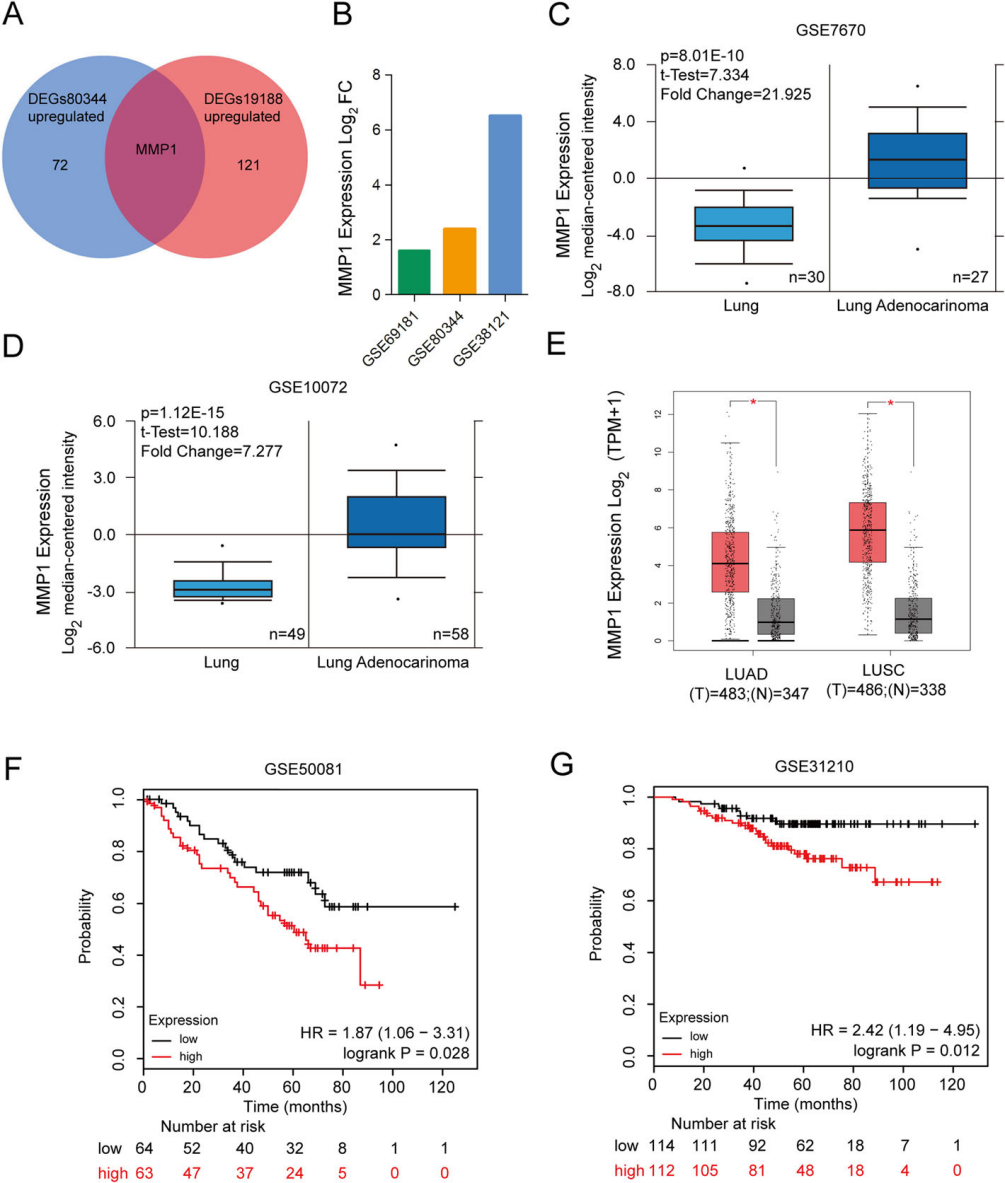
MMP1 is identified as a potential gene conferring erlotinib resistance in NSCLC. **a** Venn diagram of significantly upregulated DEGs in GSE80344 and GSE19188. **b** The expressions of MMP1were verified through datasets GSE38121 (*P* = 2.06E-8, Log_2_ FC = 6.52), GSE69181 (*P* = 2.25E-7, Log_2_ FC = 1.61) and GSE80344 (*P* = 5.67E-3, Log_2_ FC = 2.39) in erlotinib resistant NSCLC cells. The expressions of MMP1were verified through datasets **c** GSE7670 (*P* = 8.01E-10, Fold Change = 21.925) and **d** GSE10072 (*P* = 1.12E-15, Fold Change = 7.277) in NSCLC tissues and normal lung tissues. **e** Box-plot revealing that the expression levels of MMP1 is significantly different between NSCLC (LUAD and LUSC) tissues and normal lung tissues by the GEPIA2 online tool (|log_2_ (Fold change) | > 2 and *P* < 0.01). **f** and **g** Survival analysis of MMP1 in NSCLC patients was obtained from KM plotter (GSE50081, HR = 1.87 (1.06–3.31), log-rank *P* = 0.028; GSE31210, HR = 2.42 (1.19–4.95), log-rank *P* = 0.012). LUAD, lung adenocarcinoma; LUSC, lung squamous cell carcinoma

We then combined the upregulated genes of GSE19188 and GSE80344 datasets to narrow down the potential genes conferring erlotinib resistance in NSCLC and found that the expression of MMP1 was markedly increased in both datasets (Fig. 3a). To further validate the expression of MMP1 between erlotinib sensitive and resistant NSCLC cells, 3 datasets were analyzed by GEO2R (|log_2_ (Fold change) | > 1 and *P* < 0.01) (Fig. 3b). The results revealed that MMP1 was significantly upregulated in the erlotinib resistance dataset GSE38121, GSE69181 and GSE80344. And we verified that the expression of MMP1 in NSCLC tissues was significantly higher than that in normal tissues, via the datasets GSE7670 (*P* = 8.01E-10, Fold Change = 21.925) and GSE10072 (*P* = 1.12E-15, Fold Change = 7.277) in Oncomine (Fig. 3c and d). In addition, to further verify the expression level of MMP1, we utilized another online analysis tool, GEPIA2. And the results revealed that MMP1 was significantly upregulated in lung adenocarcinoma (LUAD) and lung squamous cell carcinoma (LUSC) compared with normal lung tissues (|log_2_ (Fold change) | > 2 and *P* < 0.01) (Fig. 3e). In addition, survival analysis by Kaplan-Meier plotter indicated that patients with high expression of MMP1 were associated with poor overall survival (GSE50081, HR = 1.87 (1.06–3.31), log-rank *P* = 0.028; GSE31210, HR = 2.42 (1.19–4.95), log-rank *P* = 0.012) (Fig. 3f and g).

### Validation of MMP1 hub genes related to erlotinib resistance in NSCLC

**Fig. 4:**
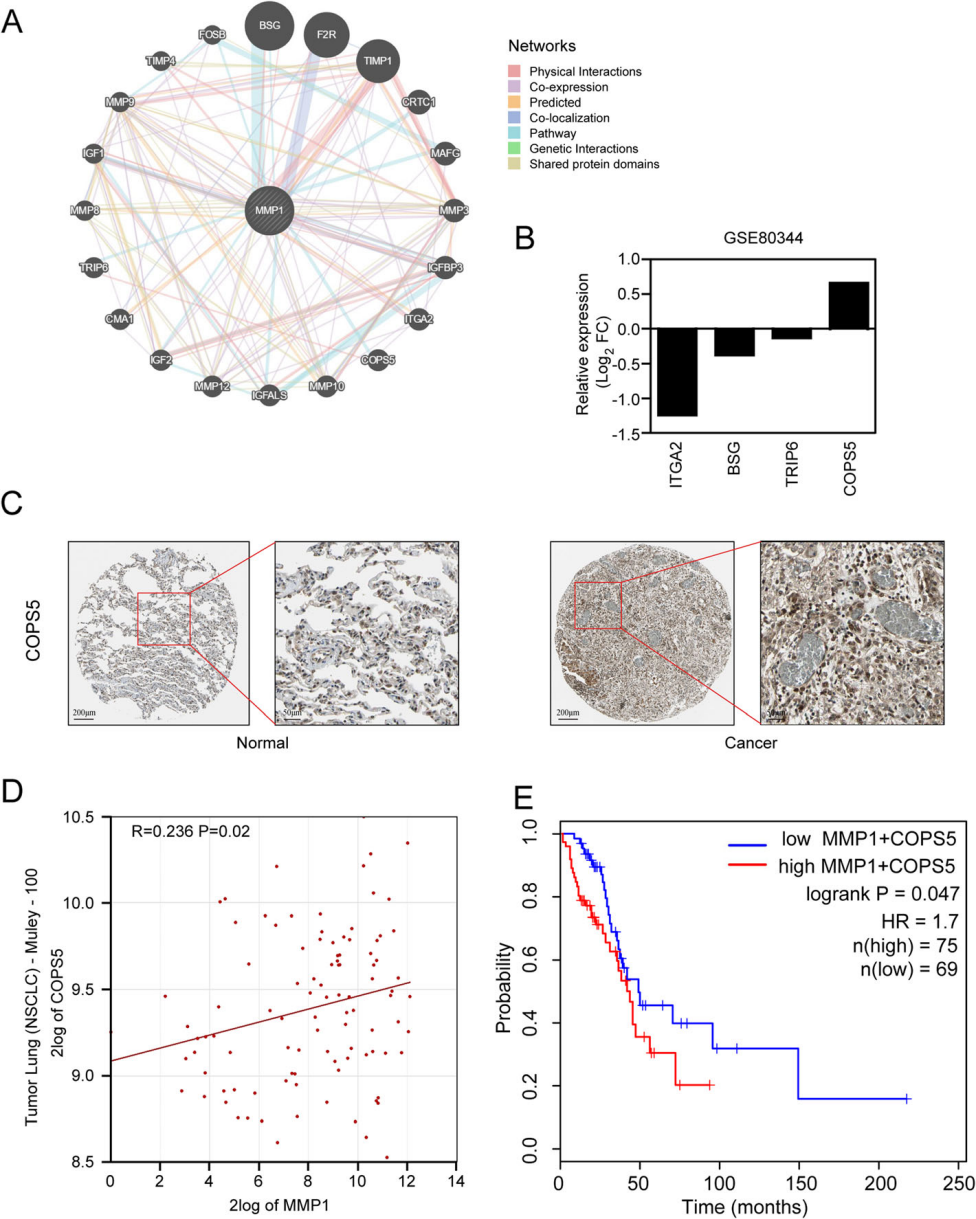
Validation of MMP1 hub genes related to erlotinib resistance in NSCLC. **a** Proteins/genes interaction network of MMP1 created by GeneMANIA. The network legends refer to the interaction types (illustrated in the networks) between proteins/genes. **b** Relative mRNA level of 4 genes interacting with MMP1 based on the microarray data of GSE80344. **c** Representative immunohistochemistry staining of COPS5 expression in normal lung tissues and NSCLC tissues. **d** Positive correlation between MMP1 and COPS5 expression in the Tumor Lung (*R* = 0.236, *P* = 0.02). **e** The prognostic value was obtained from the GEPIA2 database when both MMP1 and COPS5 were highly expressed (HR = 1.7, *P* = 0.047)

To determine how MMP1 plays functions in the erlotinib resistance of NSCLC, the GeneMANIA analysis revealed that MMP1 is closely related to 20 proteins/genes (Fig. 4a). Among these genes, four genes play crucial regulatory roles in drug resistance in a variety of cancer types. Among these four genes, Integrin subunit alpha 2 (ITGA2), Basigin (BSG), and Thyroid hormone receptor interactor 6 (TRIP6) were downregulated in the erlotinib resistance dataset GSE80344, while COP9 signalosome subunit 5 (COPS5) was upregulated in this dataset (Fig. 4b). Considering that gene expression was not always consistent with its protein level, we further analyzed the protein level of COPS5 in NSCLC tissues from the Human Protein Atlas (HPA) database. The immunohistochemical results indicated that the expression level of COPS5 exhibited significant upregulation in NSCLC tissues (Fig. 4c). Meanwhile analysis of the Tumor Lung (NSCLC)-Muley-100 dataset by R2 online tool revealed that MMP1 expression was positively correlated with COPS5 (Fig. 4d; *R* = 0.236, *P* = 0.02). And we further performed the survival analysis by GEPIA2 and found that patients with simultaneous high expression of MMP1 and COPS5 were associated with poor overall survival (HR = 1.7, log-rank *P* = 0.047) (Fig. 4e). Thus, COPS5 might be involved in MMP1-mediated erlotinib resistance.

### Validation of the Erlotinib resistance function of MMP1

**Fig. 5:**
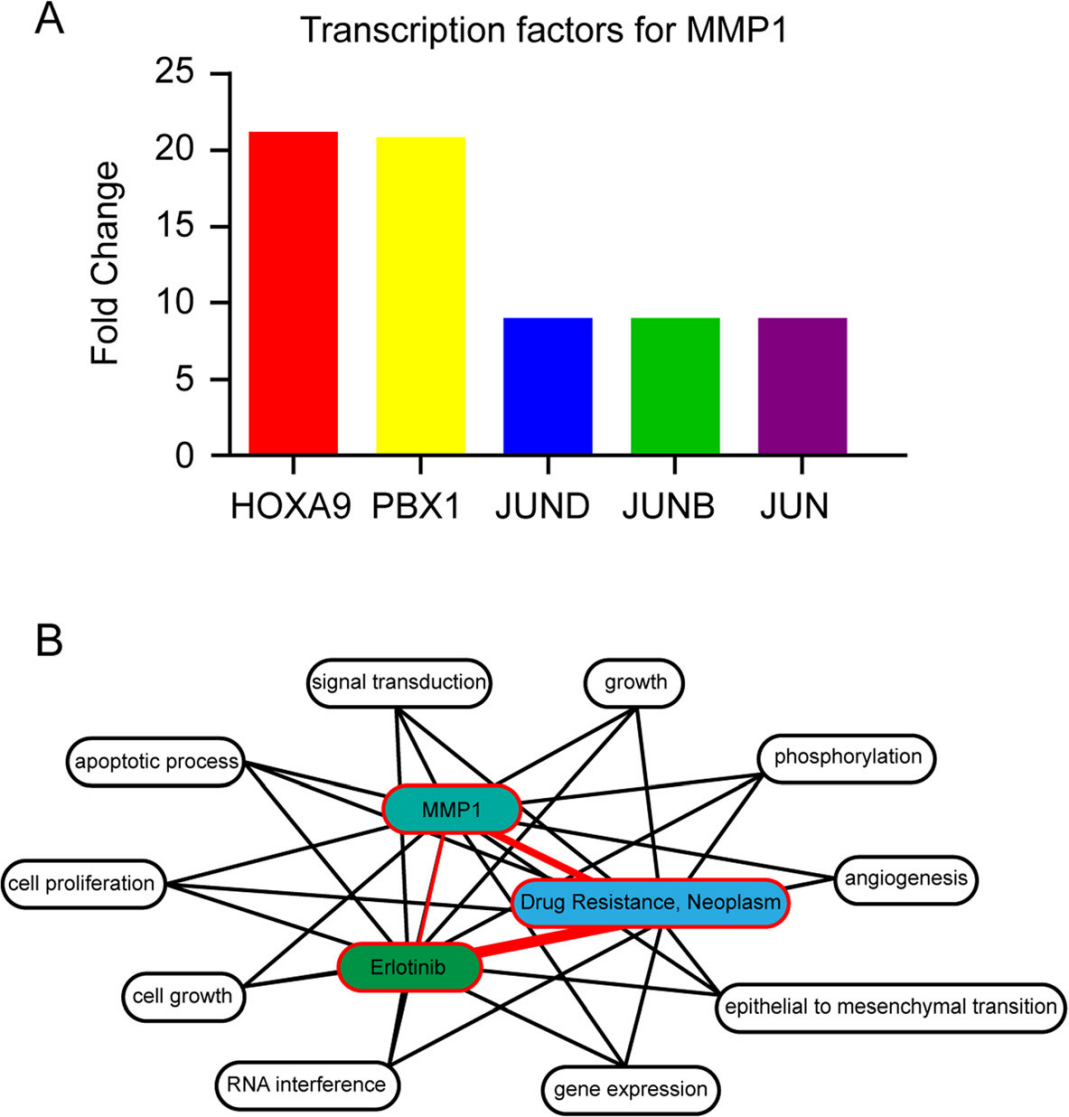
Validation of the erlotinib resistance function of MMP1. **a** Relative expression of transcription factors target MMP1 including HOXA9, PBX1, JUND, JUNB, and JUN. **b** Annotation of biological processes of MMP1 with erlotinib and drug resistance using the Coremine Medical online tool

To further investigate the mechanism of erlotinib resistance in NSCLC, the transcription factors targeting MMP1 were explored by FunRich analysis, HOXA9, PBX1, JUND, JUNB, and JUN were identified to be the top five transcription factors which targeting MMP1 (Fig. 5a). Among these transcription factors, HOXA9 [13, 36] and PBX1 [37, 38] have been reported to function as drivers in drug resistance of multiple cancer types. Finally, the biological process of MMP1 was annotated using the Coremine Medical Database. As shown in Fig. 5b, 10 biological processes were closely associated with drug resistance, which MMP1 may affect drug resistance by regulating cell growth, apoptosis, protein phosphorylation, and angiogenesis. Consequently, these results indicated that MMP1 may confer the erlotinib resistance in NSCLC through a variety of mechanisms.

## Discussion

Conventional chemotherapy and molecular-targeted therapy are currently two important strategies for NSCLC treatment [9]. Whereas, the clinical prognosis of both strategies is poor due to the subsequently acquired drug resistance, whose mechanisms have been partly reported, such as the drug target genetic mutations [39, 40]. The significance of EGFR has been identified in NSCLC, and consequently promotes the application of EGFR inhibitors [41]. Erlotinib is the first generation of EGFR inhibitors, and it has been commonly used in locally advanced or metastatic NSCLC after chemotherapy failure [10]. Although the efficacy of erlotinib in NSCLC has been confirmed, long course application of erlotinib inevitably leads to drug resistance and tumor relapse [42, 43]. At present, gene identifications conferring drug resistance through high-throughput screening methods and the design of drugs aiming at these genes may be a promising appeal in cancer treatment [8, 44]. For example, LEE011, a CDK 4/6 inhibitor, has a good inhibitory effect on the resistance caused by PI3K mutation in breast cancer [45]. In the present study, we combined an NSCLC erlotinib resistant microarray (GSE80344) with an NSCLC tissue Dataset (GSE19188), and initially identified MMP1 as a candidate gene conferring erlotinib resistance in NSCLC based on the comprehensive bioinformatics analyses.

Matrix metalloproteinases (MMPs) family is a zinc-dependent peptidase, which is responsible for the degradation of the extracellular matrix (ECM) [46]. Moreover, MMPs play a key role in tissue remodeling and regulation in various diseases containing arthritis, cirrhosis, and cancers [47, 48]. Among them, MMP1 has been proved to be highly expressed in various kinds of cancers, mainly acting to degrade type I and type III collagen in the extracellular environment [49]. It has been reported that MMP1 enhanced tumor cell invasiveness by increasing vascular endothelial growth factor (VEGF) and bone morphogenetic protein 2/4 [50–52]. In addition, Slug enhanced MMP1 transcription by directly binding to the promoter region of breast cancer cells, resulting in multiple drug resistance [53]. Additionally, it has been reported that MMP1 can be applied as a candidate therapeutic target and biomarker for drug response [17, 54, 55]. In the present study, we revealed that MMP1 was overexpressed in NSCLC tissues and erlotinib-resistant NSCLC cells, and it negatively associated with overall survival in NSCLC patients. Through using protein/gene interactions (GeneMANIA), biological process annotation (Coremine) and transcription factor analysis (FunRich), we further verified that MMP1 may confer the erlotinib resistance in NSCLC through various mechanisms. GeneMANIA results indicated that MMP1 was closely associated with 20 genes, including BSG, TRIP6, ITGA2, F2R, MMP3, COPS5, etc. And four genes (ITGA2, BSG, TRIP6, and COPS5) have been found to regulate drug resistance in different cancer models [56–59]. Especially, COPS5 was significantly upregulated in the erlotinib resistance dataset GSE80344 and NSCLC tissues. And the survival analysis indicated that patients with simultaneous high expression of MMP1 and COPS5 were associated with poor overall survival. It has been reported that COPS5 plays a crucial role in tamoxifen resistance in oestrogen receptor α (ERα) positive breast cancer patients, through regulating NCoR ubiquitination-proteasomal degradation. Other studies revealed that the expression of MMP1 was positively correlated with COPS5 [60]. These results demonstrated that COPS5 might be involved in MMP1-mediated erlotinib resistance. Several transcription factors in the epigenetic regulation of MMP1 were also screened in the present study, and two major transcription factors, HOXA9 and PBX1, were identified. Previous studies have also reported that HOXA9 and PBX1 were involved in drug resistance regulation [36, 37], suggesting that HOXA9 and PBX1 may play an important role in erlotinib resistance through regulating the transcription of MMP1. Taken together, we validated that MMP1 is a potential erlotinib resistance gene in NSCLC and could be a prognostic biomarker for erlotinib treatment.

## Conclusion

In conclusion, we demonstrated that MMP1 may confer the erlotinib resistance in NSCLC through multiple mechanisms based on comprehensive bioinformatics analyses. Our study provides a potential target for reversion and prognosis of erlotinib resistance in NSCLC, and further studies will clarify the underlying molecular mechanism by which MMP1 regulates erlotinib resistance.

## Supplementary information

**Additional file 1: Supplementary Table 1.** Identification of DEGs.

**Additional file 2: Supplementary Table 2.** GO enrichment analysis.

**Additional file 3: Supplementary Table 3.** KEGG pathway analysis.

**Additional file 4: Supplementary Table 4.** Identification of DEGs.

**Additional file 5: Supplementary Table 5.** GO enrichment analysis.

**Additional file 6: Supplementary Table 6.** KEGG pathway analysis.

## Data Availability

The datasets analyzed during the current study are available in the GEO database (https://www.ncbi.nlm.nih.gov/geo).

## Abbreviations

BP: Biological process
BSG: Basigin
CC: Cellular component
COPS5: COP9 signalosome subunit 5
DAVID: Visualization and Integrated Discovery
DEGs: Differentially expressed genes
ECM: Extracellular matrix
EGFR: Epidermal growth factor receptor
ERα: Oestrogen receptor α
FC: Fold change
GEO: Gene Expression Omnibus; GO: Gene Ontology
HPA: The Human Protein Atlas
ITGA2: Integrin subunit alpha 2
KEGG: Kyoto Encyclopedia of Genes and Genomes
KM: Kaplan-Meier
LUAD: Lung adenocarcinoma
LUSC: Lung squamous cell carcinoma
MAPK: Mitogen-activated protein kinase
MF: Molecular function
MMP1: Matrix metalloproteinase 1
NSCLC: Non-small cell lung cancer
STAT3: Signal transducer and activator of transcription 3
TRIP6: Thyroid hormone receptor interactor 6
VEGF: Vascular endothelial growth factor
