# Supplementary material for: Identification of MMP1 as a potential gene conferring erlotinib resistance in non-small cell lung cancer based on bioinformatics analyses
Source: Hereditas. 2020 Jul 23;157:32. doi: 10.1186/s41065-020-00145-x (PMC7379796; doi:10.1186/s41065-020-00145-x)
Supplement: Supplementary file 1 — Additional file 1: Supplementary Table 1. Identification of DEGs. [file 41065_2020_145_MOESM1_ESM.docx]

**Supplementary Table 1:** 308 DEGs were identified from GSE80344, including 73 upregulated genes and 235 downregulated genes in the erlotinib resistant NSCLC cells compared to normal NSCLC cells.

| DEGs | Gene names |
| --- | --- |
| Upregulated DEGs |  |
|  | GPR68, VNN1, IL1A, PTX3, SLC37A2, C1S, TNFAIP6, PAX6, ANXA10, LOC645638, PAQR5, HAVCR1, DENND2C, SLC7A2, GFPT2, LOC283050, ZEB1, CYP1B1, COL4A6, IL4I1, ZEB2, TMEM158, EMP3, TM4SF19, CEP112, SGK1, XLOC_003457, SLC16A1, CLGN, AKR1C3, ELMOD1, IL1RAPL1, FGFR1, GLIPR1, CLMP, LOC100506912, C11orf96, IL6, MMP1, G0S2, SPOCD1, FLJ31104, Q952V6, LOC645722, ARHGEF4, DOCK4, AKR1C1, COL4A5, C1R, GATA6, PYGO1, IL7, GNG4, LOC100287314, CYP1B1-AS1, ST7-AS1, CTSC, TGM2, CAPN5, SATB2, ITPR1, WDR19, MGAT4C, MGC11082, RAD21L1, TPK1, POLR3G, XLOC_l2_013513, LINC00460, TIMD4, TRAF1, EBI3, C14orf34 |
| Downregulated DEGs |  |
|  | EPHB3, FZD8, BTG2, FLJ42709, TMEM233, TM7SF2, HABP2, PIP, LOC441956, CTSH, GATA5, B3GALNT1, ALDOC, XLOC_002408, PCDHB14, ADCY4, C11orf93, IGF1, LOC100505535, SULT2B1, PRRG4, EFEMP1, EMP2, LOC100132741, XLOC_007389, FGF9, GALNT5, FAM153B, CA11, CCNI2, PRINS, TMEM139, TMEM61, APCDD1, CYP1A1, FAAH, CAPN12, TRIB2, SFTPB, C10orf81, MB, KLK4, AQP3, TSPAN13, LOC100505946, BMP7, NOTCH3, LRCH2, PPP1R14D, DNM3, KRT6A, NEURL1B, FAR2, EPS8L1, TFAP2D, LGALS9, SPTLC3, HPGD, NYNRIN, COL9A3, FHL2, FADS2, XLOC_010995, LOC730102, CEACAM1, NLRP12, FAM153A, NTM, CST6, ADORA1, TCEA3, PDE9A, GJB5, XLOC_l2_011910, FRMD3, IRX4, GPR133, PCDHB11, PCDHB9, SYTL1, BST2, VSNL1, ABCA4, LOC729860, ARHGAP44, XLOC_002771, JAKMIP2, MYO1D, SYTL2, RASSF10, KLF13, XLOC_l2_007543, FGD3, SEMA5B, MSMB, PCDHB16, CRYM, GCNT3, ZNF711, TFF3, PMEPA1, SPINK2, PCDHB8, MAGIX, LIMCH1, SESN3, ST6GALNAC2, C19orf46, TNFSF10, CYP2B6, SYNPO, FMO5, GPM6B, NDN, XLOC_006350, DLL1, XLOC_001243, ATP10A, C14orf159, ABCA13, EFHD1, RASD1, MFSD4, CEACAM5, ZPLD1, EPB41L4A, LOC440335, PELI2, SBK1, FGFBP1, SLC40A1, SLC13A3, ENTPD3, RORC, FAM49A, PCSK9, KRTAP4-1, TMEM125, LMO3, RIPK3, CRLF1, XLOC_l2_009301, MCF2L, GPR64, SLC16A9, SLC44A4, ENPP3, DENND2A, ARHGAP4, NELL1, C13orf15, SIDT1, TSPAN1, TLE2, PCDH20, MPV17L, GABBR2, APOD, POF1B, CKMT1A, NDNF, ID4, KLHDC9, LOC100129480, SDPR, C19orf81, ICAM2, KCNMB4, ATP2C2, LY75, PRR15L, NOSTRIN, PLEKHG1, LRRTM1, C9orf152, FGF19, HPN, ST6GALNAC1, C17orf28, DOK7, KLK7, RNF43, NAPSA, MUC1, XLOC_008823, INPP5D, DMBT1, C1orf116, PRODH, CYB5R2, CAPN8, ERN2, NEBL, CST1, CXCL17, KAL1, SYT13, KLK8, C2orf54, TNS4, KRT4, NMNAT2, LOC389023, RARRES3, TMC4, AUTS2, BCAS1, PLA2G10, SPTSSB, SLITRK6, VSTM2L, FAM174B, ZBED2, IFI27, KLK6, ANO1, MIR205HG, RNASE1, HOPX, NPTX2, SPTB, CELF2, CRIP1, SLCO2A1, PROC, AGR2, SLC34A2, KLK5, VTCN1, CEACAM3, PHACTR3, CEACAM7, INHBB, SPOCK2, CEACAM6 |

Note: The DEGs are listed from the largest to the smallest of fold changes.
