# Supplementary material for: Identification of MMP1 as a potential gene conferring erlotinib resistance in non-small cell lung cancer based on bioinformatics analyses
Source: Hereditas. 2020 Jul 23;157:32. doi: 10.1186/s41065-020-00145-x (PMC7379796; doi:10.1186/s41065-020-00145-x)
Supplement: Supplementary file 2 — Additional file 2: Supplementary Table 2. GO enrichment analysis. [file 41065_2020_145_MOESM2_ESM.docx]

**Supplementary Table 2:** GO enrichment analysis results of upregulated DEGs in DEG80344 (P<0.01 and |logFC|≥2).

| Category | #Pathway ID | Pathway description | Genes | P-value |
| --- | --- | --- | --- | --- |
| GOTERM_BP_DIRECT | GO:0060445 | branching involved in salivary gland morphogenesis | FGFR1, IL6, TGM2 | 5.08E-04 |
| GOTERM_BP_DIRECT | GO:0042574 | retinal metabolic process | AKR1C3, CYP1B1, AKR1C1 | 6.09E-04 |
| GOTERM_BP_DIRECT | GO:0006954 | inflammatory response | TNFAIP6, IL6, VNN1, GPR68, PTX3, IL1A | 0.0062138 |
| GOTERM_BP_DIRECT | GO:0019221 | cytokine-mediated signaling pathway | IL6, TGM2, IL1A, EBI3 | 0.0077615 |
| GOTERM_BP_DIRECT | GO:0006955 | immune response | IL6, IL7, CTSC, C1R, ZEB1, IL1A | 0.0095618 |
| GOTERM_BP_DIRECT | GO:0071395 | cellular response to jasmonic acid stimulus | AKR1C3, AKR1C1 | 0.0123305 |
| GOTERM_BP_DIRECT | GO:0006959 | humoral immune response | IL6, IL7, EBI3 | 0.0134655 |
| GOTERM_BP_DIRECT | GO:0030574 | collagen catabolic process | MMP1, COL4A6, COL4A5 | 0.0167769 |
| GOTERM_BP_DIRECT | GO:0021902 | commitment of neuronal cell to specific neuron type in forebrain | SATB2, PAX6 | 0.0214804 |
| GOTERM_BP_DIRECT | GO:0044598 | doxorubicin metabolic process | AKR1C3, AKR1C1 | 0.0245119 |
| GOTERM_MF_DIRECT | GO:0003682 | chromatin binding | SATB2, RAD21L1, GATA6, PYGO1, PAX6, ZEB1 | 0.00637 |
| GOTERM_MF_DIRECT | GO:0047086 | ketosteroid monooxygenase activity | AKR1C3, AKR1C1 | 0.00903662 |
| GOTERM_MF_DIRECT | GO:0047718 | indanol dehydrogenase activity | AKR1C3, AKR1C1 | 0.00903662 |
| GOTERM_MF_DIRECT | GO:0018636 | phenanthrene 9,10-monooxygenase activity | AKR1C3, AKR1C1 | 0.012031 |
| GOTERM_MF_DIRECT | GO:0047115 | trans-1,2-dihydrobenzene-1,2-diol dehydrogenase activity | AKR1C3, AKR1C1 | 0.012031 |
| GOTERM_MF_DIRECT | GO:0001227 | transcriptional repressor activity, RNA polymerase II transcription regulatory region sequence-specific binding | PAX6, ZEB2, ZEB1 | 0.0137193 |
| GOTERM_MF_DIRECT | GO:0005125 | cytokine activity | IL6, IL7, IL1A, EBI3 | 0.0160891 |
| GOTERM_MF_DIRECT | GO:0004032 | alditol:NADP+ 1-oxidoreductase activity | AKR1C3, AKR1C1 | 0.020961 |
| GOTERM_MF_DIRECT | GO:0016655 | oxidoreductase activity, acting on NAD(P)H, quinone or similar compound as acceptor | AKR1C3, AKR1C1 | 0.0239200 |
| GOTERM_MF_DIRECT | GO:0004252 | serine-type endopeptidase activity | CTSC, C1R, C1S, MMP1 | 0.0417169 |
| GOTERM_CC_DIRECT | GO:0005576 | extracellular region | FGFR1, IL6, IL7, GLIPR1, IL4I1, C1R, C1S, PTX3, MMP1, EBI3, COL4A6, IL1A, COL4A5 | 0.002644 |
| GOTERM_CC_DIRECT | GO:0005587 | collagen type IV trimer | COL4A6, COL4A5 | 0.017974 |
| GOTERM_CC_DIRECT | GO:0005581 | collagen trimer | MMP1, COL4A6, COL4A5 | 0.031486 |
| GOTERM_CC_DIRECT | GO:0005886 | plasma membrane | CEP112, FGFR1, SGK1, CLMP, GPR68, ITPR1, PAQR5, DOCK4, WDR19, SLC16A1, SLC7A2, GLIPR1, TGM2, VNN1, GNG4, EMP3, IL1RAPL1, EBI3 | 0.09767 |
