# Supplementary material for: Identification of MMP1 as a potential gene conferring erlotinib resistance in non-small cell lung cancer based on bioinformatics analyses
Source: Hereditas. 2020 Jul 23;157:32. doi: 10.1186/s41065-020-00145-x (PMC7379796; doi:10.1186/s41065-020-00145-x)
Supplement: Supplementary file 3 — Additional file 3: Supplementary Table 3. KEGG pathway analysis. [file 41065_2020_145_MOESM3_ESM.docx]

**Supplementary Table 3:** KEGG pathway analysis results of upregulated DEGs in DEG80344 (P<0.01 and |logFC|≥2).

| Category | #Pathway ID | Pathway description | Genes | P-value |
| --- | --- | --- | --- | --- |
| KEGG_PATHWAY | hsa04151 | PI3K-Akt signaling pathway | FGFR1, SGK1, IL6, IL7, GNG4, COL4A6, COL4A5 | 0.0022428 |
| KEGG_PATHWAY | hsa05133 | Pertussis | IL6, C1R, C1S, IL1A | 0.0033527 |
| KEGG_PATHWAY | hsa05200 | Pathways in cancer | TRAF1, FGFR1, IL6, GNG4, MMP1, COL4A6, COL4A5 | 0.0043053 |
| KEGG_PATHWAY | hsa00140 | Steroid hormone biosynthesis | AKR1C3, CYP1B1, AKR1C1 | 0.02295 |
| KEGG_PATHWAY | hsa05222 | Small cell lung cancer | TRAF1, COL4A6, COL4A5 | 0.0463485 |
| KEGG_PATHWAY | hsa04640 | Hematopoietic cell lineage | IL6, IL7, IL1A | 0.0483285 |
| KEGG_PATHWAY | hsa05323 | Rheumatoid arthritis | IL6, IL1A, MMP1 | 0.0493301 |
| KEGG_PATHWAY | hsa05146 | Amoebiasis | IL6, COL4A6, COL4A5 | 0.0685946 |
