# Supplementary material for: Identification of MMP1 as a potential gene conferring erlotinib resistance in non-small cell lung cancer based on bioinformatics analyses
Source: Hereditas. 2020 Jul 23;157:32. doi: 10.1186/s41065-020-00145-x (PMC7379796; doi:10.1186/s41065-020-00145-x)
Supplement: Supplementary file 4 — Additional file 4: Supplementary Table 4. Identification of DEGs. [file 41065_2020_145_MOESM4_ESM.docx]

**Supplementary Table 4：**369 DEGs were identified from GSE19188 including 122 upregulated genes and 247 downregulated genes in NSCLC tissues compared to normal lung tissues.

| DEGs | Gene names |
| --- | --- |
| Upregulated DEGs |  |
|  | KRT6A, MMP12, GREM1, AKR1B10, GJB2, CXCL13, NUF2, COL11A1, TPX2, MMP1, PBK, UHRF1, TTK, GINS1, UBE2C, UBE2T, ANLN, CTHRC1, DLGAP5, BUB1B, TOP2A, RRM2, CCNB1, COL10A1, FOXM1, MELK, CDC20, PRC1, CDKN3, HJURP, ADAMDEC1, KIF4A, KRT5, MAGEA6, FAM64A, CEP55, KRT6B, TUBB2B, PCP4, SPP1, NCAPG, NEK2, PSAT1, NMU, KIF20A, EXO1, GPR87, SCG5, HMMR, TRIP13, SPINK1, RMI2, DSP, SLC6A8, DTL, SPRR1B, KIAA0101, CKAP2L, NTS, PRAME, RAD51AP1, S100A2, KIF2C, RAD54L, KIF11, HS6ST2, SULF1, SHCBP1, MAD2L1, CDCA7, NUSAP1, GJB6, PLK1, KIF14, FAM83D, OIP5, CDCA3, PYCR1, MKI67, NCAPG2, CENPK, KIF15, CCNA2, EZH2, KRT15, CTSV, LOC101929272, PPAP2C, KRT14, DEPDC1, CDC6, DSG3, CENPE, GALNT14, CDC45, E2F8, HES6, HMGB3, KRT16, RFC4, DNMT3B, MDK, HMGB3P1, THBS2, CST4, ATAD2, BIRC5 /// EPR-1, GPX2, DUXAP10, SPC25, LOC344887, CRABP2, UBE2S, DEPDC1B, STIL, PITX1, KIF18B, UGT8, MMP3, C12orf56, KIF23, CYP24A1 |
| Downregulated DEGs |  |
|  | TMEM100, GKN2, SFTPC, MAMDC2, AGER, CLDN18, SLC6A4, SFTPD, CPB2, WIF1, CYP4B1, SCGB1A1, FABP4, LRRK2, TNNC1, PPBP, NAPSA, MCEMP1, CA4, SCGB3A2, C4BPA, INMT, FCN3, PEBP4, OLR1, CHRDL1, SCN4B, ZNF385B, GDF10, EDNRB, C16orf89, AQP4, FOLR1, FMO2, CFD, AOC3, HSD17B6, NOSTRIN, NDNF, IGSF10, SDPR, AGR3, ADIRF, PLA2G1B, ADH1B, MT1M, FAM150B, MFAP4, SOX7, CACNA2D2, OGN, FOSB, SCGB3A1, ATP13A4-AS1, TYRP1, NR3C2, NTN4, EMCN, ACADL, LOC101926959, SFTA1P, ADAMTS8, PCAT19, SFTA3, FAM107A, ANKRD29, ABCA3, ADRB2, LAMP3, LPL, GPX3, SFTPB, SFTA2, C2orf40, FHL1, EP300-AS1, SELENBP1, TCF21, FAM189A2, ANXA3, CNTN6, PGC, LINC00968, CDH5, GPR116, ANGPTL1, HPGD, SOSTDC1, SUSD2, GPR133, HHIP, AGTR1, S1PR1, FOXF1, ACKR4, LYVE1, CLIC3, LRRN3, ZBTB16, CCDC68, C7, AQP1, MRC1, GPM6A, CAV1, KCNT2, SELE, IL6, HPGDS, MARCO, FLRT3, TGFBR3, C1QTNF7, PCOLCE2, VGLL3, CPA3, RAMP3, ICAM4, CAPSL, PPP1R14A, KLB, LOC100996760, SLC46A2, NRGN, JAM2, FGG, IL33, NCKAP5, CYS1, CFP, TPPP3, SLC34A2, SLCO2A1, FILIP1, KIAA1462, SLIT2, CALCRL, LRRC36, CSRNP1, SLC6A14, DMBT1, VIPR1, STEAP4, FIBIN, SLC26A9, SLPI, MYOCD, SEMA3G, FBLN5, GIMAP6, WISP2, ITIH5, FAM162B, LOC101928612, PPARG, FCN1, VSIG4, COLEC12, ZBED2, CLIC5, EMP2, PLLP, WFDC1, CD52, FCGR3B, COL6A6, CCL23, VEPH1, ZFP36, SCN7A, SLC1A1, GPIHBP1, BMP5, FLJ35700, KANK3, ACKR1, TMEM178A, CMTM2, MYZAP, IRX1, LRP2, SLC14A1, TCEAL2, HBB, USP44, CCL2, RETN, RTKN2, MS4A7, DRAM1, KLF4, CYP3A7-CYP3AP1, ALOX5AP, MAL, SGMS2, FBP1, SDR16C5, CA2, STX11, SERTM1, SHISA3, C20orf85, LRRC32, BCHE, CYP2B7P, FGR, CD69, MNDA, SELP, HLF, FXYD1, BMP2, C14orf132, S100A4, ATP1A2, AGTR2, MS4A15, PRICKLE2, FOS, GPR146, SYNE1, SOX17, NEXN, COL21A1, HYAL1, LOC286189, ALDH2, TMEM125, CLEC1A, ESAM, PTPRB, CCBE1, C16orf54, PDZD2, P2RY14, FGFBP2, CPAMD8, PLA1A, CRTAM, MYCT1, SCEL, SOCS2, PTX3, SYNC, KLRF1, TPSB2, TRPC6 |

Note: The DEGs are listed from the largest to the smallest of fold changes.
