# Supplementary material for: Identification of MMP1 as a potential gene conferring erlotinib resistance in non-small cell lung cancer based on bioinformatics analyses
Source: Hereditas. 2020 Jul 23;157:32. doi: 10.1186/s41065-020-00145-x (PMC7379796; doi:10.1186/s41065-020-00145-x)
Supplement: Supplementary file 5 — Additional file 5: Supplementary Table 5. GO enrichment analysis. [file 41065_2020_145_MOESM5_ESM.docx]

**Supplementary Table 5:** GO enrichment analysis results of upregulated DEGs in DEG19188 (P<0.01 and |logFC|≥2).

| Category | #Pathway ID | Pathway description | Genes | P-value |
| --- | --- | --- | --- | --- |
| GOTERM_BP_DIRECT | GO:0007018 | microtubule-based movement | KIF14, KIF23, KIF2C, KIF4A, KIF11, KIF15, KIF18B, CENPE, KIF20A | 1.21E-08 |
| GOTERM_BP_DIRECT | GO:0000281 | mitotic cytokinesis | KIF23, PLK1, NUSAP1, ANLN, KIF20A | 1.33E-05 |
| GOTERM_BP_DIRECT | GO:0007059 | chromosome segregation | SPC25, KIF11, OIP5, HJURP, NEK2, BIRC5 | 1.45E-05 |
| GOTERM_BP_DIRECT | GO:0090307 | mitotic spindle assembly | KIF11, NEK2, TPX2, BIRC5 | 7.23E-04 |
| GOTERM_BP_DIRECT | GO:1904668 | positive regulation of ubiquitin protein ligase activity | PLK1, CDC20, UBE2S | 0.001034774 |
| GOTERM_BP_DIRECT | GO:0031145 | anaphase-promoting complex-dependent catabolic process | CDC20, UBE2C, UBE2S | 0.001034774 |
| GOTERM_BP_DIRECT | GO:0007094 | mitotic spindle assembly checkpoint | MAD2L1, PLK1, BUB1B | 0.003177443 |
| GOTERM_BP_DIRECT | GO:0070979 | protein K11-linked ubiquitination | UBE2C, UBE2S, UBE2T | 0.007163907 |
| GOTERM_BP_DIRECT | GO:0000070 | mitotic sister chromatid segregation | PLK1, NEK2, KIF18B | 0.007163907 |
| GOTERM_BP_DIRECT | GO:0001578 | microtubule bundle formation | PRC1, PLK1, KIF20A | 0.008814459 |
| GOTERM_MF_DIRECT | GO:0003777 | microtubule motor activity | KIF14, KIF23, KIF2C, KIF15, KIF18B, CENPE, KIF20A | 1.98E-06 |
| GOTERM_MF_DIRECT | GO:0005524 | ATP binding | KIF14, KIF23, CDC6, KIF4A, KIF11, NEK2, KIF15, ATAD2, KIF18B, TTK, CENPE, PBK, UBE2C, RAD54L, KIF2C, RFC4, PLK1, TOP2A, UBE2S, MELK, UBE2T, TRIP13, KIF20A | 2.39E-06 |
| GOTERM_MF_DIRECT | GO:0008574 | ATP-dependent microtubule motor activity, plus-end-directed | KIF14, KIF4A, KIF11, KIF18B | 9.30E-05 |
| GOTERM_MF_DIRECT | GO:0016887 | ATPase activity | KIF14, KIF23, KIF2C, KIF15, ATAD2, KIF20A | 3.84E-04 |
| GOTERM_MF_DIRECT | GO:0061631 | ubiquitin conjugating enzyme activity | UBE2C, UBE2S, UBE2T | 0.009775 |
| GOTERM_MF_DIRECT | GO:0004222 | metalloendopeptidase activity | MMP3, ADAMDEC1, MMP12, MMP1 | 0.028385 |
| GOTERM_MF_DIRECT | GO:0003682 | chromatin binding | EXO1, CDC45, KIAA0101, ATAD2, TOP2A, UBE2T | 0.042177 |
| GOTERM_MF_DIRECT | GO:0031625 | ubiquitin protein ligase binding | UBE2C, UBE2S, UBE2T | 0.053498 |
| GOTERM_MF_DIRECT | GO:0005198 | structural molecule activity | KRT5, KRT15, KRT14, DSP | 0.055369 |
| GOTERM_MF_DIRECT | GO:0005509 | calcium ion binding | DSG3, SULF1, MMP3, THBS2, MELK, MMP12, MMP1, S100A2 | 0.061085 |
| GOTERM_CC_DIRECT | GO:0005871 | kinesin complex | KIF14, KIF23, KIF2C, KIF4A, KIF11, KIF15, KIF18B, KIF20A | 2.78E-08 |
| GOTERM_CC_DIRECT | GO:0030496 | midbody | KIF14, KIF23, PRC1, PLK1, NEK2, BIRC5, KIF20A | 1.72E-05 |
| GOTERM_CC_DIRECT | GO:0000922 | spindle pole | CDC6, KIF11, PLK1, NEK2, TPX2 | 6.52E-04 |
| GOTERM_CC_DIRECT | GO:0000777 | condensed chromosome kinetochore | SPC25, HJURP, BIRC5 | 0.001257 |
| GOTERM_CC_DIRECT | GO:0005876 | spindle microtubule | KIF11, PLK1, NUSAP1, BIRC5 | 0.001324 |
| GOTERM_CC_DIRECT | GO:0005654 | nucleoplasm | EXO1, KIF23, CDC6, CYP24A1, PRC1, DTL, FOXM1, CRABP2, TPX2, ATAD2, CDC20, ANLN, DEPDC1, RAD54L, HS6ST2, DNMT3B, TOP2A, CCNA2 | 0.004887 |
| GOTERM_CC_DIRECT | GO:0051233 | spindle midzone | KIF14, CDC6, PLK1 | 0.005197 |
| GOTERM_CC_DIRECT | GO:0005874 | microtubule | KIF14, KIF23, TPX2, KIF18B, KIF20A | 0.005757 |
| GOTERM_CC_DIRECT | GO:0000775 | chromosome, centromeric region | OIP5, HJURP, BIRC5 | 0.006568 |
| GOTERM_CC_DIRECT | GO:0005634 | nucleus | HMGB3, NEK2, FOXM1, E2F8, KIAA0101, KRT5, OIP5, NCAPG2, PCP4, HJURP, TOP2A, DLGAP5, NUF2, ATAD2, RMI2, BIRC5, CENPK, UBE2C, CDKN3, CCNB1, KRT15, RRM2, KRT14, DSP, UBE2S, MELK, UBE2T | 0.007512 |
