# Supplementary material for: Identification of MMP1 as a potential gene conferring erlotinib resistance in non-small cell lung cancer based on bioinformatics analyses
Source: Hereditas. 2020 Jul 23;157:32. doi: 10.1186/s41065-020-00145-x (PMC7379796; doi:10.1186/s41065-020-00145-x)
Supplement: Supplementary file 6 — Additional file 6: Supplementary Table 6. KEGG pathway analysis. [file 41065_2020_145_MOESM6_ESM.docx]

**Supplementary Table 6:** KEGG pathway analysis results of upregulated DEGs in DEG19188 (P<0.01 and |logFC|≥2).

| Category | #Pathway ID | Pathway description | Genes | P-value |
| --- | --- | --- | --- | --- |
| KEGG_PATHWAY | cfa04110 | Cell cycle | CCNB1, CDC6, CDC45, MAD2L1, PLK1, TTK, BUB1B, CDC20, CCNA2 | 2.40E-07 |
| KEGG_PATHWAY | cfa04914 | Progesterone-mediated oocyte maturation | CCNB1, MAD2L1, PLK1, CCNA2 | 0.011356 |
| KEGG_PATHWAY | cfa04512 | ECM-receptor interaction | THBS2, COL11A1, SPP1, HMMR | 0.011717 |
| KEGG_PATHWAY | cfa05206 | MicroRNAs in cancer | KIF23, CYP24A1, EZH2, DNMT3B | 0.039896 |
| KEGG_PATHWAY | cfa05323 | Rheumatoid arthritis | MMP3, CTSV, MMP1 | 0.073873 |
